# Properties of Bovine Collagen as Influenced by High-Pressure Processing

**DOI:** 10.3390/polym15112472

**Published:** 2023-05-26

**Authors:** Milan Houška, Aleš Landfeld, Pavla Novotná, Jan Strohalm, Monika Šupová, Tomáš Suchý, Hynek Chlup, Jan Skočilas, Jan Štípek, Margit Žaloudková, Miloslav Šulc

**Affiliations:** 1 Food Research Institute Prague, Radiová 1285/7, Hostivař, 10200 Prague, Czech Republic; ales.landfeld@vupp.cz (A.L.); pavla.novotna@vupp.cz (P.N.); jan.strohalm@vupp.cz (J.S.); miloslav.sulc@vupp.cz (M.Š.); 2 Institute of Rock Structure and Mechanics, Czech Academy of Sciences, V Holešovičkách 41, 18209 Prague, Czech Republic; supova@irsm.cas.cz (M.Š.); suchy@irsm.cas.cz (T.S.); zaloudkova@irsm.cas.cz (M.Ž.); 3 Department of Process Engineering, Faculty of Mechanical Engineering, Czech Technical University in Prague, Technická 4, 16000 Prague, Czech Republic; hynek.chlup@fs.cvut.cz (H.C.); jan.skocilas@fs.cvut.cz (J.S.); jan.stipek@fs.cvut.cz (J.Š.)

**Keywords:** bovine collagen, high-pressure processing, physical properties, structure properties

## Abstract

The physical properties and structure of collagen treated with high-pressure technologies have not yet been investigated in detail. The main goal of this work was to determine whether this modern gentle technology significantly changes the properties of collagen. High pressure in the range of 0–400 MPa was used, and the rheological, mechanical, thermal, and structural properties of collagen were measured. The rheological properties measured in the area of linear viscoelasticity do not statistically significantly change due to the influence of pressure or the duration of pressure exposure. In addition, the mechanical properties measured by compression between two plates are not statistically significantly influenced by pressure value or pressure hold time. The thermal properties T_on_ and ∆H measured by differential calorimetry depend on pressure value and pressure hold time. Results from amino acids and FTIR analyses show that exposure of collagenous gels to high pressure (400 MPa), regardless of applied time (5 and 10 min), caused only minor changes in the primary and secondary structure and preserved collagenous polymeric integrity. SEM analysis did not show changes in collagen fibril ordering orientation over longer distances after applying 400 MPa of pressure for 10 min.

## 1. Introduction

Collagen has been extensively studied as the dominant component of the extracellular matrix of animal and human bodies. Its chemistry and use were well described by Lasek [1]. The collagen protein family is characterized by great diversity of structure, occurrence, and function. So far, 29 types of collagen proteins have been classified [2].

Collagen consists of amino acids bound together to form an α helix, such that left-handed polyproline II-type helices join to form a single right-handed triple helix of elongated fibrils [3]. This unique fibrous structure predisposes collagen to various applications. Collagen-based materials are widely used in reconstructive medicine, pharmaceuticals, cosmetics, tanning, and the food industry [4]. The tissue regeneration capabilities of collagen-based biomaterials represent the future of medical soft and hard tissue development [5] (e.g., in the fields of regeneration in the treatment of periodontal disease [6], dental implants [7], and repair of osteochondral defects [8]). Collagen’s unique structure makes it useful as a protein carrier in drug delivery systems, especially in treating cancer and genetic diseases [9].

Collagen and its derivatives, such as gelatin and hydrolyzed peptides, are consumable. Thanks to various functional and bioactive properties, they have great potential in the food industry (e.g., as food additives, food packaging and preservation materials, and functional food ingredients) [10].

Over the past decades, hydrostatic pressure has been used to analyze the structural properties and phase behavior of protein systems [11]. As a relatively new technology, ultra-high pressure (UHP) has attracted widespread interest in specific biotechnologies, such as food production [12,13]. UHP can also be an attractive alternative to traditional food preservation (heat pasteurization or sterilization). Its limited effects on covalent bonds and α-helix or β-sheet structures result in minimal nutritional value and sensory quality changes. UHP is a non-thermal food treatment that subjects liquid or solid foods to pressures between 50 and 1000 MPa [14]. In addition, high pressure (HP) can enhance several processing operations, such as freezing, thawing, and extraction, thus providing new processing options.

Collagen is an integral part of raw food materials (such as muscle tissue from mammals, fish, and poultry); collagen can also be used as an edible film or coating. Therefore, studies of the effect of UHP on collagen structure and mechanical properties are of great value for using UHP technology in the food industry. UHP technology, in combination with chemical reagents, is widely used for obtaining gelatin from collagen [15,16]. The use of high pressure for the homogenization of micro/nano collagen fibers is also possible [17].

Under high hydrostatic pressure, proteins can show changes in their native structure analogous to changes that occur at high temperatures. This effect relates to reversible and irreversible changes in the protein’s native structure [18]. However, high pressure can also leave parts of the molecule unchanged, indicating that HP denaturation mechanisms are substantially different from those of high temperature [19]. Tauscher [20] reported that the secondary and tertiary/quaternary structures of most proteins are compromised by high pressures of 400 and 200 MPa, respectively.

Collagen can be extracted from natural products that remain after food production (e.g., feathers, animal hair, animal skin, crustacean shells, fish scales, and bones). However, the integrity of the extracted collagen-based materials (i.e., structural stability and resistance to various treatments (chemical, thermal, irradiation)) is influenced by the collagen source (animal genus, sex, age, breeding, etc.), the extraction method, and other treatments [21].

The few papers published on UHP treatment of collagen have dealt with porcine (pig) [22] and bullfrog [23] collagen. One of the most important studies of UHP (up to 200 MPa) on porcine collagen structure (concentration of 1.5 mg/mL) was performed by Potekhin et al. [22]. Their results showed that the thermal stability of collagen was significantly improved with increasing pressure, which is quite different from the effects on globular proteins, where structural stability is reduced after UHP. However, their study was based on microcalorimetric analyses done during real-time ultra-high-pressure treatment and a theoretical analysis derived from other proteins.

Nan et al. [23] systematically analyzed the molecular structures and properties of a bullfrog collagen solution (5 mg/mL) using UHP up to 500 MPa. They used SDS-polyacrylamide gel electrophoresis, Fourier-transformed infrared spectroscopy, and circular dichroism measurements to characterize changes in collagen structure. They showed that at pressures less than 400 MPa, the dominant forces were perpendicular to the triple helix, while at pressures above 400 MPa, the dominant forces were along the axis of the helix.

This work aimed to demonstrate experimentally whether the structure and physical properties (viscoelastic, mechanical, and thermal) of bovine collagen are affected by UHP treatment at varying pressures up to 400 MPa and by different holding times (5 and 10 min). Collagenous gel subjected to UHP was based on type I bovine COL isolated from calf skin homogenized in water (7.44 wt.%), which is used to produce sausage casings.

This study aimed to analyze the changes in chemical composition (i.e., the concentration of water and amino acids) and structural parameters (i.e., the effect on the triple-helix and β-sheet, the ratio of mature/immature crosslinks, and fiber orientation). Additionally, we studied changes in the rheological, mechanical, and thermal properties of the collagenous gels after UHP. This study of collagen structures and physical properties after UHP has significant reference value for the use of UHP in the food industry and other applications.

## 2. Materials and Methods

### 2.1. Material

The studied collagenous material consisted of natural bovine collagen (type I) supplied by DEVRO, Ltd. (Jilemnice, Czech Republic), a company specializing in producing collagen casings. The original solution contained 7.44 wt.% of collagen. The collagen was extracted from mechanically and chemically pretreated bovine skin. Samples (≈100 g) were taken from a bag weighing approximately 10 kg. The samples were placed in bags made of double-layered polyethylene/polyamide (hereafter PE/PA). They were then vacuum sealed with a weld.

### 2.2. Apparatus

**Figure 1:**
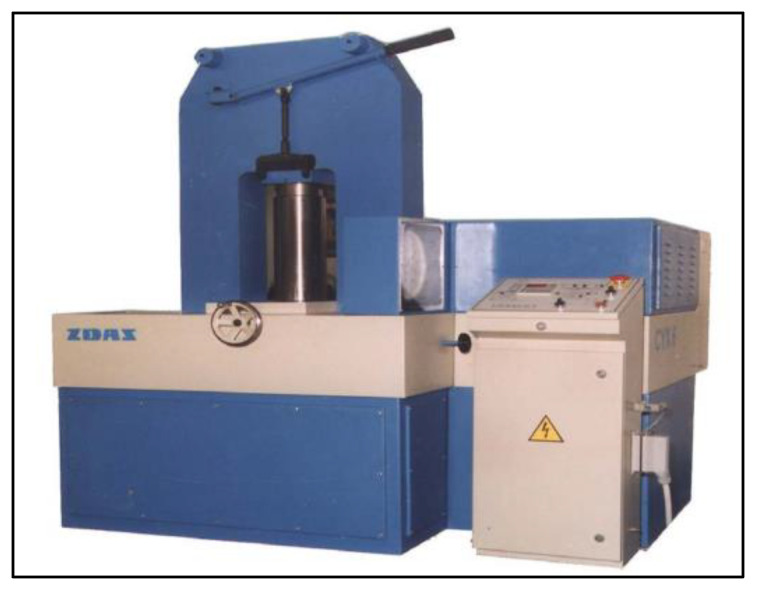
CYX 6/103 high-pressure isostatic press made by Žďas joint-stock company with a chamber volume of 2 L.

An isostatic press, CYX 6/103, manufactured by Žďas, a joint-stock company (Žďár nad Sázavou, Czech Republic), was used to treat collagen samples sealed in vacuum bags (see Figure 1). The device is equipped with a high-pressure chamber with a volume of 2 L, where the pressure of the drinking water can reach up to 450 MPa. Pressure can be applied for different holding times (5–15 min); plastic bottles or PE/PA or PE/Al bags serve as packaging. The treatment takes place in the final package, which must be made of suitable plastic packaging (i.e., it must prevent tap water, under high pressure, from penetrating the packaging or the product from escaping the packaging and returning to its original shape after depressurization).

### 2.3. Pressurizing Samples

The prepared packaged samples were placed individually in the pressurization chamber of the device, which was previously filled with tap water at a temperature of about 17 °C. The chamber was closed, and an automated system was started to achieve the preselected pressures and durations. All samples were successively treated at pressures of 200, 300, and 400 MPa with holding times of 5 and 10 min. During pressurization, the sample is heated, but due to the capacity of the metal chamber, the energy is dissipated during the pressurization period. After depressurization, the sample cools down.

The temperature of the medium (i.e., the pressurizing water in the chamber) was measured before and after each treatment of each sample. After pressure treatment, the temperatures of the sample were also measured. The initial temperature of the pressurized water in the chamber was 20 °C during the first series of treatments. After treatment, the temperatures of the pressurized water in the chamber ranged from 18.5 to 19 °C. The temperatures of the samples after pressure treatment ranged from 17.3 to 17.9 °C.

During the second series of treatments of the same collagen material, the starting temperature of the pressurized water in the chamber was 19.3 °C. After treatment, the temperature of the pressurized water in the chamber ranged from 16.5 to 18.1 °C. The temperature of the samples after pressure treatment was constant (14.5 °C). It follows from the mentioned temperatures that the thermal transformation of collagen into gelatin could not occur during the pressure treatments used in our study.

### 2.4. Determination of Dry Matter

The dry matter of the collagen samples was determined by their weight in a hot air oven at a temperature of 105 °C after 24 h. Three weighings were always carried out, and the result was averaged.

### 2.5. Determination of pH

A Testo type 206 pH meter (Testo SE & Co. KGaA, Titisee-Neustadt, Germany) was used to measure pH. This device simultaneously measures the temperature of the material.

### 2.6. Rheological Measurement

Oscillatory rheological properties were measured using a method similar to that reported by Landfeld et al. [24]. The measurement was taken on a Haake RS150 Rheostress rheometer (Thermo Fisher Scientific, Life Technologies Czech Republic Ltd., Prague, Czech Republic). Frequency oscillations ranged from 0.1 to 1.778 Hz, with a relative deformation of 0.04. The range was determined in advance based on an amplitude test in the linear viscoelastic region so that the elastic modulus does not change to this value while increasing the amplitude of oscillations at a constant frequency of 1 Hz. A plate-plate geometry was used for the measurements. The diameter of the top plate was 35 mm, and the gap between the plates was 2 mm. The measurement temperature was a constant 10 °C. Each measurement was performed five times; each repetition was with a new sample. The elastic modulus (G′) and loss modulus (G′′) and the phase angle δ were measured for each sample.

### 2.7. Measurement of Mechanical Properties

A TA/XT2 Texturometer from Stable Micro Systems (Surrey, UK), with a force-measuring probe capable of handling up to 50 N (manufacturer’s specification 5 kg) was used for the measurements. Collagen in the form of a cylinder with a diameter of 15 mm and an initial height of 20 mm was placed between two circular plates and compressed at a speed of 1 mm/second so that the relative deformation did not exceed 0.3. Forces (F) were converted to tension (Ϭ) by dividing by the area of the face of the roller, whose starting radius was R_0_. A correction was made for the increasing diameter of the cylinder due to compression, which is valid under the assumption of the conservation of the cylinder volume:

σ = F/[π⋅R_0_^2^⋅(H_0_/(H_0_ − x⋅H_0_))] (1)

where x = y/H_0_, and “y” represents the axial deformation of the cylinder. Nine measurements were acquired for each pressurized sample time.

Our data can be described by almost any hyperelastic model (e.g., the Holzapfel–Gasser–Ogden three-parameter model (HGO) [25]). For uniaxial loading where deformation is less than 0.3, the HGO model can be simplified to a two-parameter model:

(2) $$\sigma =E\varepsilon +18k_{1}\varepsilon^{3}$$

In the case of very small deformations *ε* or for *k*_1_ = 0, the model reduces to the form of Hooke’s model, where *E* represents the modulus of elasticity (or Young’s modulus when there is uniaxial tension). We have successfully used this approach to model the mechanical behavior of collagen gels in our previous studies (e.g., under uniaxial tension [26]). For the identification of the HGO model and for calculating the associated statistics, the data were processed using MATLAB (The MathWorks, Inc., Natick, MA, USA).

The cross-comparison of the collagen gels was carried out by comparing the HGO models using qualitative cluster analysis and, in particular, by comparing the potential changes in the stiffness characteristics of the collagen by comparing the compression moduli. The moduli of elasticity in compression were determined as the slope of the initial linear part of the stress-strain relationship (*ε* 0.05–0.15) for each sample (i.e., *n* = 9 in each group).

### 2.8. Measurement of Thermal Properties

A Perkin Elmer Diamond DSC (PE Systems Ltd., Prague, Czech Republic) differential scanning calorimetry device with an Intracooler 2P temperature unit was used for thermal measurements. The samples were placed in aluminum capsules with a collagen weight of 10–50 micrograms. The measurement was carried out while increasing the temperature of the capsule. Each measurement was repeated at least five times. The device software evaluates, from the measured energy consumption required to heat the sample, the temperature at the beginning of the peak T_on_, the temperature at the peak T_peak_, the height of the peak H_peak_, and the value of the area under the peak (i.e., ΔH), which is proportional to the energy of the reaction caused by heating the sample.

### 2.9. Determination of Total Water Content

The determination of the free water and the interstitial water (directly bound to the triple-helix) [27] was performed using the ISO 6496:1983 standard (Animal feedstuffs—Determination of the moisture content), that is, drying to 160 ± 2 °C for 4 h (dryer—Memmert GmbH + Co. KG, Büchenbach, Germany); scales—Mettler-Toledo Ltd. (Prague, Czech Republic).

### 2.10. Infrared Spectrometry (FTIR)

The secondary structure of the collagenous materials was analyzed using infrared spectrometry with an iS50 infrared spectrometer (Nicolet Instrument, Madison, WI, USA) in reflection mode (ATR) with a diamond crystal (GladiATR, PIKE Technologies, Madison, WI, USA). Collagens were scanned in a freeze-dried state after 400 MPa of pressure in a gel state for 5 and 10 min. The materials were measured 20 times (*n* = 20) to verify collagen homogeneity. ATR-FTIR spectra were recorded in the middle-spectral range of 4000–400 cm^−1^ via 64 scans at a resolution of 4 cm^−1^. Acquired spectra were processed using OMNIC version 9 software (Thermo Scientific, Madison, WI, USA). The areas of the amide I bands were deconvoluted using the same software and statistically evaluated.

### 2.11. Amino Acids by HPLC-DAD

Amino acid analysis was based on Agilent procedures (Amino Acid Analysis—Application Compendium), with some modifications [28].

#### 2.11.1. Chemicals and Solutions

Mobile phase A: aqueous buffer containing 10 mM Na_2_HPO_4_ (anhydrous), 10 mM Na_2_B_4_O_7_ (decahydrate), 5 mM NaN_3_, pH set to 8.2 (with conc. HCl), and filtered through 0.2 µm nylon filter. Mobile phase B: acetonitrile, methanol, water (45:45:10, v/v/v). Injection diluent: 100 mL mobile phase A and 0.4 mL H_3_PO_4_ (85%). Ortho-phthalaldehyde (OPA), 9-fluorenylmethyl chloroformate (FMOC), and the borate buffer (0.4 M in water, pH 10.2) provided by Agilent (in a kit). For hydrolysis, 0.1 M HCl, 6M HCl was purged with N_2_ for at least 30 min. Needle wash: mobile phase B. Reconstitution solution: 500 µmol/L IS (sarcosine, norvaline) in 0.05 M HCl. Calibration solutions (in 0.05 M HCl): 21 amino acids at 90, 225, and 900 µmol/L containing IS (500 µmol/L) prepared from an Agilent AA standard kit according to instructions and stored at −20 °C. Milli-Q HPLC-grade water (>18 MΩ). All chemicals were HPLC or ACS grade and were purchased from Merck Life Science Ltd. (Prague, Czech Republic), Lach-ner, Ltd. (Neratovice, Czech Republic), Agilent (Santa Clara, CA, USA, or Linde Gas join stock company (Prague, Czech Republic).

#### 2.11.2. Collagen Hydrolysis

A 0.5 g sample of bovine collagen was weighed into a 15 mL Hungate anaerobic glass culture tube, 10 mL 6M HCl (purged with N_2_) was added, and the headspace was flushed with N_2_, vortexed (IKA^®^-Werke GmbH & Co. KG, Staufen, Germany) for 30 s, and put into a laboratory oven (BINDER GmbH, Tuttlingen, Germany) at 110 °C for 20 h, with occasional inversions to mix the contents. After hydrolysis, samples were cooled down to room temperature and vortexed for 30 s. Then, 300 µL of hydrolysate was evaporated in an HPLC vial under N_2_ at 60 °C for 15 min (BT Lab Systems, Saint Louis, MO, USA). To the dry residue, 1 mL of reconstitution solution was added, vortexed for 1 min, and filtered through a 0.2 µm nylon syringe filter into an HPLC glass vial with a silicone/PTFE screw cap (Chromservis Ltd., Prague, Czech Republic). Each sample was prepared in ten replicates.

#### 2.11.3. HPLC-DAD

An Agilent (Santa Clara, CA, USA) type 1260 Infinity II HPLC consisting of a degasser, column oven (40 °C), autosampler (5 °C), and a DAD detector equipped with an Agilent Poroshell HPH-C18, 3 × 100 mm, 2.7 µm column with a guard column (HPH-C18, 3 × 5 mm; 2.7 µm), were used. Derivatization vials: borate buffer, OPA, FMOC, and injection diluent. On-line derivatization (autosampler) procedure: valve to bypass, needle wash for 10 s, wait 0.3 min, draw 2.5 µL borate buffer, draw 1 µL sample, needle wash 5 s, mix 3.5 µL in air 5 times, wait 0.2 min, draw 0.5 µL OPA, mix 4 µL in air 10 times, draw 0.4 µL FMOC, mix 4.4 µL in air 10 times, draw 32 µL from injection dilution sol., mix 20 µL in air eight times, needle wash 10 s, inject, wait 0.4 min, valve bypass. Gradient (only mobile phase B given): 0 min 2%, 0.45 min 2%, 13.5 min 57%, 13.6 min 100%, 17.6 min 100%, 18 min 2%, 23 min 2%. Mobile phase flow: 0.62 mL/min. A four-point calibration (including origin) using the two IS was drawn with R^2^ > 0.999 for each amino acid. Wavelengths: 338 nm for OPA derivatives (10 nm bw. 390 nm ref. and 20 nm ref. bw.) and 262 nm for FMOC derivatives (16 nm bw. 324 nm ref. and 8 nm ref. bw.).

### 2.12. Scanning Electron Microscopy (SEM) and the Characterization of the Orientation of the Collagen Fibrils

To visualize the morphology and orientation of the collagen fibrils of collagenous materials before and after pressure application, they were scanned using a STEM Apreo S2 microscope (Thermo Scientific, Madison, WI, USA), in high vacuum mode with an Everhart–Thornley detector in secondary electron mode at 5 and 10 keV. Several randomly selected samples were examined for inhomogeneity. The materials were fixed using Palay solution [29] for 2 h at room temperature, followed by overnight fixation at 4 °C. The fixed samples were washed in a phosphate buffer and an ethanol and acetone dehydration series using a Leica EM TP tissue processor (Specion Ltd., Prague, Czech Republic) and dried on a Leica EM CPD300 critical point drier. The dried samples were then mounted on stubs using carbon adhesive stickers and sputter coated with Pt in an Ar atmosphere using the Leica EM ACE600 coating system. Overview electron micrographs were then taken at magnifications of 5000× and 10,000×.

### 2.13. Statistical Analysis

Statistical analysis (water content, FTIR, amino acid composition, and mechanical properties during compression) was performed in GraphPad Prism (ver. 9.5.0 (730), GraphPad Software, San Diego, CA, USA). The normality of the data was verified using Shapiro–Wilk’s test and the construction of Q-Q plots. The homoscedasticity was verified using Levene’s and Bartlett’s tests. Non-parametric analysis was employed since the assumption of normality or homoscedasticity was violated. The Kruskal–Wallis test for multiple comparisons was performed with a subsequent post hoc test based on Dunn’s test. The Mann–Whitney test was performed to compare differences between two independent samples. Statistical significance was accepted at *p* ≤ 0.05.

### 2.14. Rheological Measurement

**Figure 2:**
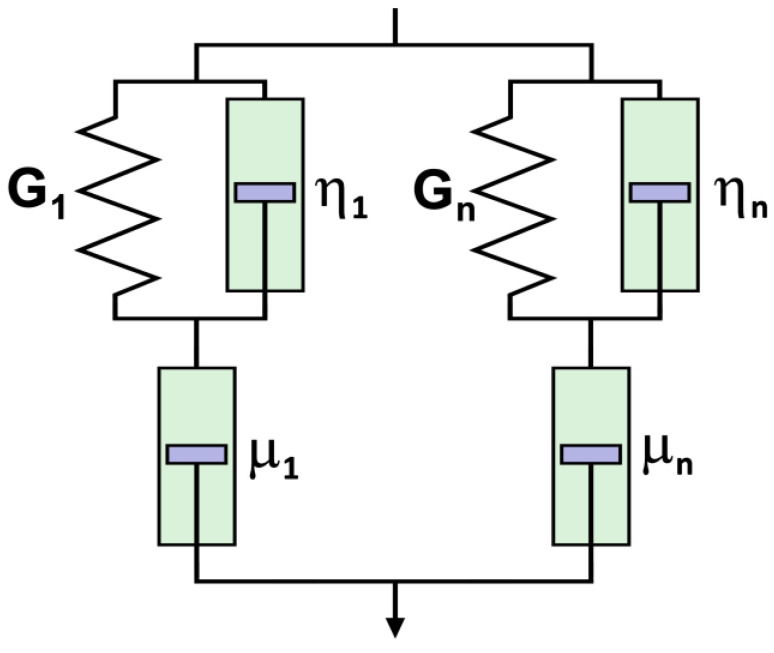
Combined Kelvin–Voigt–Maxwell model (adapted from Refs. [24,31]).

All rheological parameters were measured repeatedly for pressurized collagen samples. Arithmetic means and standard deviations were calculated for all parameters. These values were plotted in all figures (bar segments represent mean values, and abscissas represent confidence intervals). Statistical evaluation of the data was performed using analysis of variance and statistical QC Expert 3.1 software (TriloByte Statistical Software, s.r.o., Pardubice, Czech Republic). For collagen, the following factors were investigated: pressure levels of 0, 200, 300, and 400 MPa, with 5 and 10 min holding times for each pressure level. The oscillation frequency was applied in the 0.1–1.778 Hz range, corresponding to an angular velocity *ω* of 0.628–11.168 rad/s. All measurements were made at a temperature of 10 °C. The observed “real” parts G′ (storage moduli), that is, the “real” part of the complex modulus of elasticity G and the “imaginary” part G′′ (loss moduli), were correlated with the angular velocity *ω* using the following linear viscoelasticity model of combined Kelvin–Voigt–Maxwell models (Barnes [30]), as seen in Figure 2 and Equations (3) and (4).

(3) $$G^{\prime }=\sum_{i=1}^{n}\frac{G_{i}{\left(\omega {µ}_{i}\right)}^{2}}{{G_{i}}^{2}+\omega^{2}{\left({µ}_{i}+{ƞ}_{i}\right)}^{2}}$$

(4) $$G^{\prime \prime }=\sum_{i=1}^{n}{µ}_{i}\omega \frac{\left({µ}_{i}+{ƞ}_{i}\right){}_{i}\omega^{2}+G_{i}^{2}}{{G_{i}}^{2}+{\omega^{2}\left({µ}_{i}+{ƞ}_{i}\right)}^{2}}$$

Remark: µ*_i_* = *G_i_⋅*τ*_i_*

Viscosity η and the complex modulus of elasticity in shear G represent a parallel damper-spring combination (Kelvin Voigt model), while the viscosity µ represents series-connected dampers. A special case of this general viscoelastic model is the Maxwell model for zero damping viscosities (η*_i_* = 0). The usual four-parameter Maxwell model (*n* = 2, η*_i_* = 0) represents an excellent approximation of the storage modulus G′ but cannot describe the loss modulus G′′ due to the asymptotic properties at high frequencies (i.e., all Maxwellian G′′ terms approach zero at ω → ∞).

Therefore, a combined three-parameter Kelvin–Voigt–Maxwell model using non-zero parallel viscosity was tested as an alternative η*_i_* for *n* = 1 (or a six-parameter model for *n* = 2). The parameters of the combined models could be adjusted to describe the plateau and the growing region (i.e., “G”).

Since elastic properties prevailed for small deformations in the tested collagen samples (G′ > G′′), only G′ data and a simple Maxwell model were used. Parameters for the two terms of the Maxwell model G_1_, G_2_, µ_1_, and µ_2_ were found using non-linear regressions of the G′ data using DataFit software version 6.1.10 (Oakdale Engineering, Pittsburgh, PA, USA) and Equation (3) simplified for η_1_ = η_2_ = 0 and *n* = 2. Parameters of the Maxwell model G_1,2_ and μ_1,2_ were correlated relative to pressure P_I_ and holding time D_I_. Relationships were examined using DataFit statistical software version 6.1.10. Parameters were correlated using linear relationships, as follows:

G_1_ = a_1_ + b1⋅DI + c1⋅PI (5)

G_2_ = d_2_ + e2⋅DI + f2⋅PI (6)

Statistical significance was assessed by comparing correlation coefficients and critical values for this parameter at a significance of α = 0.05 and degrees of freedom d_f_ = the number of experimental points minus the number of model parameters: 7 − 3 = 4; r_crit, 4_ = 0.811. Data on critical values of correlation coefficients were taken from a publication by Štěpánek [32].

### 2.15. Measurement of Mechanical Properties

The data of axial stresses, which depend on relative strain ϵ, was approximated by the least squares regression method using MATLAB (The MathWorks, Inc., Natick, MA, USA). software using Equation (2). As described above, this software determined the numerical values for coefficients E and k1 of Equation (2).

### 2.16. Measurement of Thermal Properties

From the data of nine replicates, mean values, standard deviations, and confidence intervals at the 0.05 significance level were calculated for all tested collagen samples. In addition, all thermal properties data were subjected to Analysis of Variance (ANOVA) (QC Expert Software, TriloByte Statistical Software, Ltd., Pardubice, Czech Republic) to determine whether there was a statistically demonstrable dependence of the measured thermal properties on the amount of applied pressure and duration of pressure.

## 3. Results

### 3.1. Dry Matter Content and pH

Collagen dry matter was determined using the mass method at 105 °C for 24 h; the average value was 7.44%. Samples were treated with 400 MPa of pressure and had a holding time of 10 min and a pH of 2.13; measurements were taken at 9.7 °C.

### 3.2. Rheological Properties

**Figure 3:**
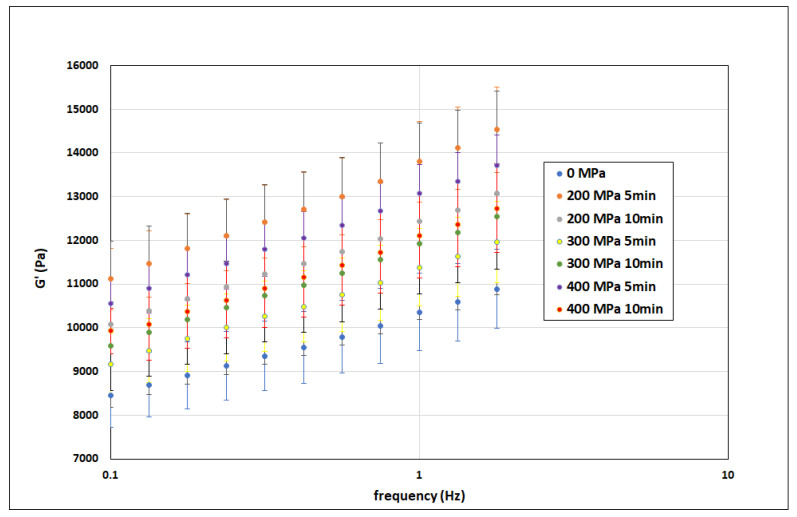
Experimental data of the storage modulus of elasticity G′ as a function of oscillation frequency and pressure treatment parameters (confidence intervals are marked).

Figure 3 presents all the measured data of the elastic modulus of elasticity G′ as a function of the oscillation frequency (the confidence intervals, determined from repeated measurements for given pressure treatment parameters, are marked).

**Figure 4:**
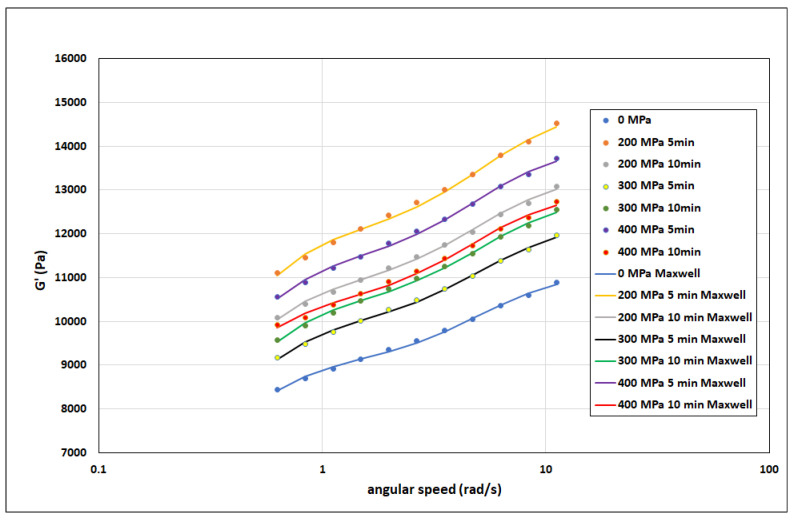
Experimental data and regression curves of the modulus of elasticity G′ as a function of angular velocity and pressure treatment parameters.

Figure 4 presents all the elastic modulus G data as a function of the angular velocity for pressure values and duration. The data are fitted by regression curves corresponding to a simplified Kelvin–Voigt model: Equation (3) for η_1,2_ = 0. The figure shows that the elastic modulus G′ grows with increasing angular velocity of oscillations. It is also evident that the simplified model we used does an excellent job of describing the experimental data for the individual parameters of pressure treatment.

#### 3.2.1. The Results of Evaluating the Parameters of the Simplified Maxwell Model (Equation (3))

The results from repeated measurements of the modulus G′ for all samples (i.e., for all pressure treatment methods, including untreated samples) were evaluated using Equation (3), and the parameters determined G_1_. G_2_, τ_1_, and τ_2_. Numerical values for these parameters, including calculated values µ_1_ and µ_2_, are presented in Table 1. This table shows the values of the correlation coefficients determined for the experimental data and valid for the individual samples (i.e., individual treatment methods). The total data for five repeated measurements of each sample were 55 (11 frequencies × 5 repetitions). The number of parameters of the model (i.e., Equation (3)) was 4. Therefore, the number of degrees of freedom is 55 − 4 = 51. For this value, r_crit,50_ = 0.273. By comparing this value with the r data in Table 1, it is clear that Equation (3) almost perfectly describes the individual measured data for a given pressure-treated sample; this is also evident in Figure 4.

#### 3.2.2. Results of Parameter Evaluation of Correlation Equations (5) and (6)

The results of the numerical evaluation of the correlation Equations (5) and (6) are shown in Table 2 and Table 3. It is clear that, apart from the constants a_1_ and d_2_, the other parameters are statistically insignificant since *p* > 0.05 demonstrates the independence of G_1_ and G_2_ from pressure holding times. For Equation (5), the value of the multiple correlation coefficient was r = 0.447. If we compare this value with the required value of the correlation coefficient of 0.811, it is evident that the G_1_ parameter does not depend on pressure or holding time. For Equation (6), the value of the multiple correlation coefficient was r = 0.488. If we compare this value with the required value of the correlation coefficient, 0.811, the G_2_ parameter also does not depend on pressure or holding time.

### 3.3. Mechanical Properties

**Figure 5:**
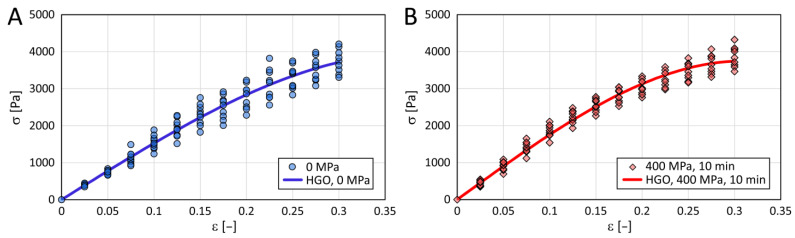
Stress-strain data for untreated collagenous gel (**A**) and gel treated at 400 MPa with a holding time of 10 min (**B**) (two extreme conditions). The colored lines represent modified Holzapfel–Gasser–Ogden (HGO) models.

In Figure 5, we present an example of the experimental data of stress as a function of relative deformation when compressing a cylinder shape of untreated collagen gel and gel treated with a pressure of 400 MPa and a holding time of 10 min. These parameters applied to the gels essentially represent the two extreme conditions in our experiment. The data were fitted with a simplified HGO model by Equation (2). Table 4 shows the identified parameters of the HGO model with coefficients of determination.

**Figure 6:**
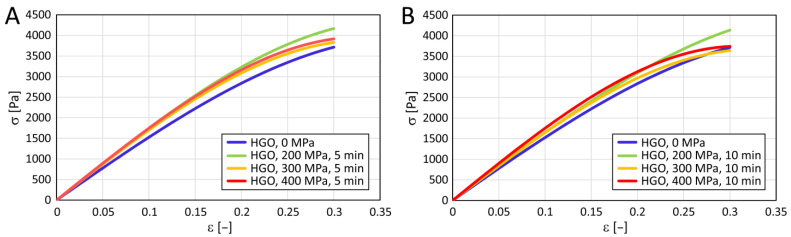
A comparison of the HGO models for untreated collagenous gel and gels treated at different pressures with a holding time of 5 (**A**) or 10 (**B**) minutes. HGO model parameters are listed in Table 4.

**Figure 7:**
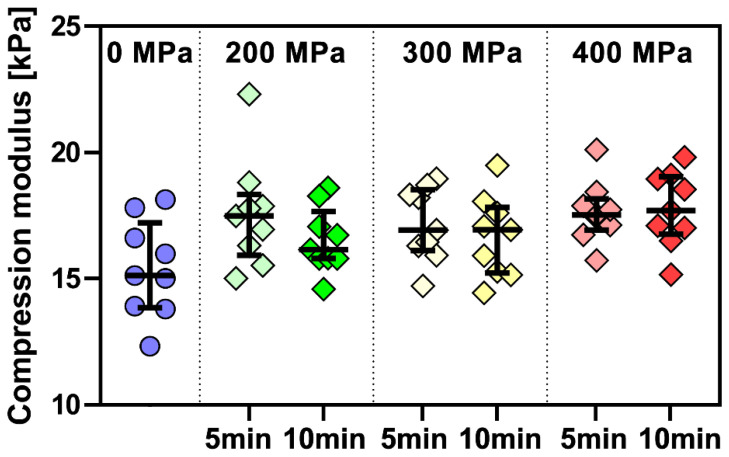
Compression modulus of collagenous gels before and after the treatment at different pressures (200, 300, and 400 MPa) with different holding times (5 and 10 min). Based on the Kruskal–Wallis test and the subsequent Dunn’s multiple comparison tests of moduli of collagen gels in all conditions, it is impossible to reject the null hypothesis (i.e., medians are equal) at the chosen significance level of 0.05 (*n* = 9).

The cross-comparison of the collagen gels is illustrated in Figure 6. Qualitative data evaluation was done by comparing the HGO models using cluster analysis, and quantitative evaluation by comparing the possible changes in their stiffness characteristics. The compression moduli of each group are shown in Figure 7. No statistically significant differences emerge from the multiple comparisons performed.

### 3.4. Thermal Properties

The evaluated thermal properties of pressure-treated collagen were subjected to an analysis of variance to determine whether pressure and holding time have a statistically demonstrable effect on thermal properties. The results of this statistical analysis are shown in Table 5.

It is clear from Table 5 that the temperature at the beginning of the thermal reaction caused by heating the T_on_ collagen sample is statistically demonstrably dependent on the pressure and the duration. The same conclusion can be drawn regarding parameter ΔH.

Parameter T_peak_ shows a statistically significant dependence on the pressure but is independent of holding time. On the other hand, parameter H_peak_ does not depend on the pressure but statistically significantly depends on the holding time.

**Figure 8:**
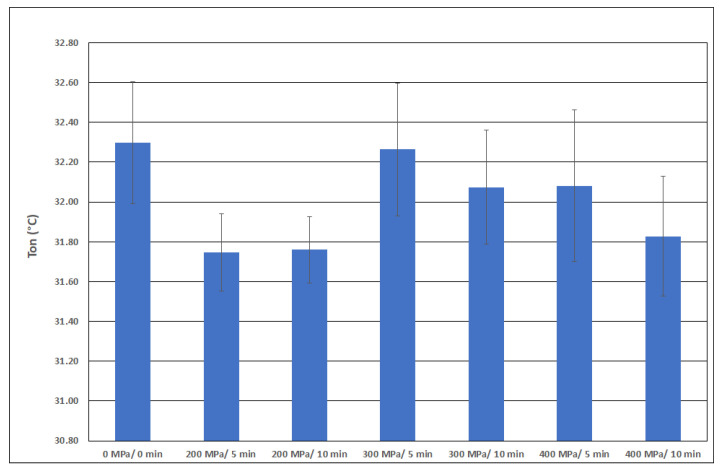
Peak onset T_on_ as a function of pressure.

**Figure 9:**
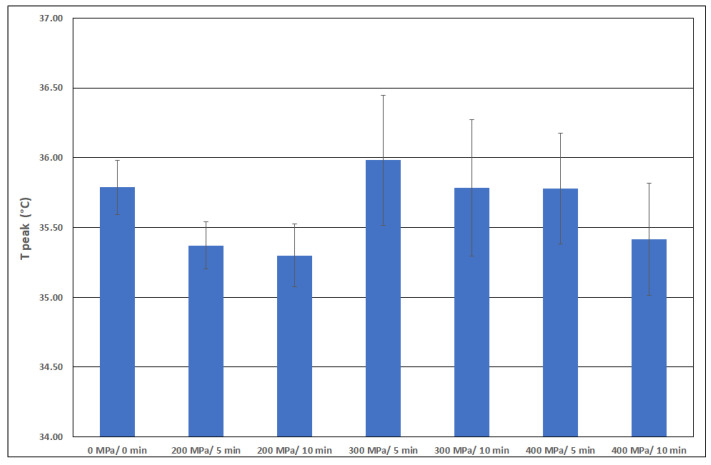
Peak temperature T_peak_ as a function of pressure.

**Figure 10:**
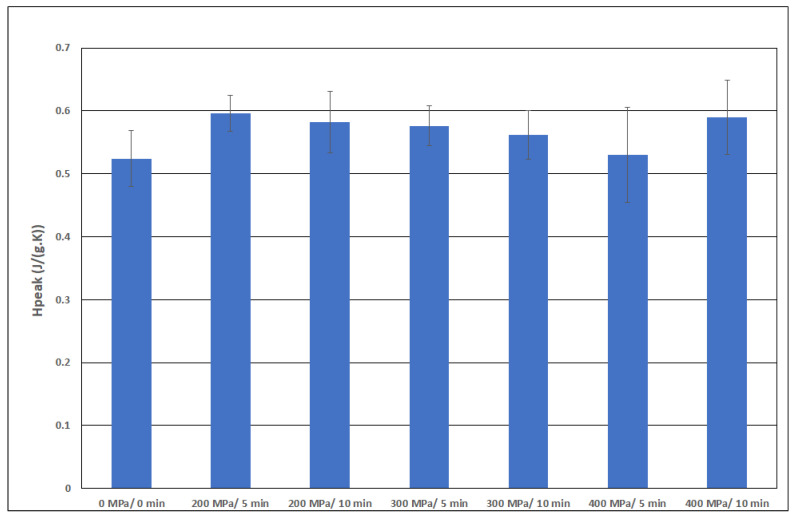
Peak height H_peak_ as a function of pressure.

**Figure 11:**
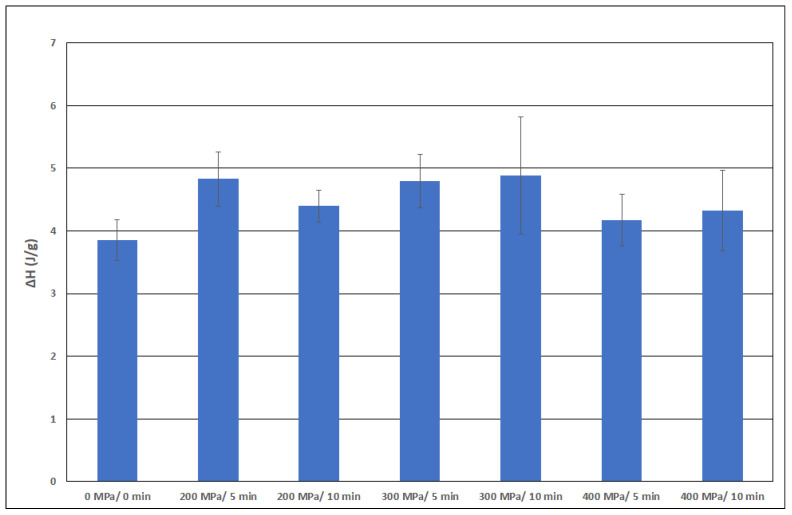
Parameter ΔH as a function of pressure.

Figure 8, Figure 9, Figure 10 and Figure 11 show the average values of all thermal properties depending on the pressure treatment parameters. The confidence intervals of these mean values at a significance level of 0.05 are also presented. Based on these intervals, it is possible to determine whether the thermal properties of the individual samples are statistically different from each other.

### 3.5. Determination of Total Water Content

**Figure 12:**
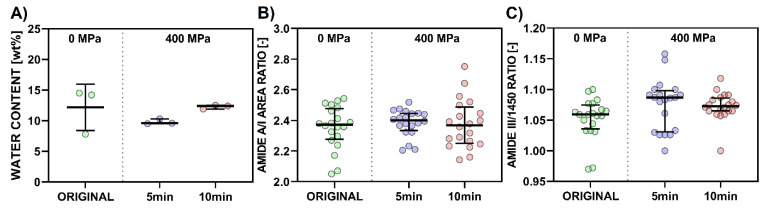
Scatter plot of (**A**) “TOTAL WATER CONTENT,” (**B**) “AREA RATIO A/I,” and (**C**) “INTENSITY RATIO AMIDE III/1450” with arithmetical mean and standard deviation for (**A**) and with medians and interquartile range for (**B**) and (**C**). Note that *p*-values less than or equal to 0.05 (Dunn’s multiple comparison test; *n* = 3 for (**A**), *n* = 20 for (**B**) and (**C**)) are displayed for the comparisons of the mean rank of each data set with the mean rank of every other data set.

Total water content, that is, free and interstitial (directly bound to the collagen triple-helix) water, even in a freeze-dried state, as determined by weight loss, is shown in Figure 12A. No statistically significant differences were seen in holding times compared to the original collagenous materials.

### 3.6. Infrared Spectrometry

**Figure 13:**
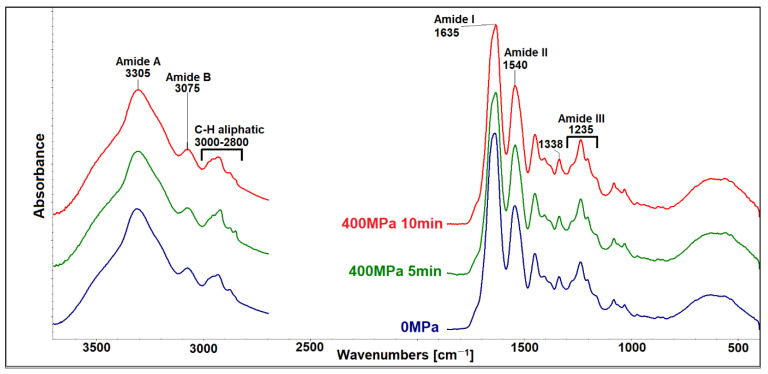
Comparisons of the infrared spectra before and after application of 400 MPa for 5 and 10 min.

ATR-FTIR enables the interpretation of changes in the secondary structure of collagen matrices after 400 MPa for 5 and 10 min. The comparison of all studied collagenous materials is shown in Figure 13.

The secondary protein structure can be described by five amidic bands in FTIR spectra [33]. The band at ~3305 cm^−1^ belongs to amide A, incorporating N-H stretching and several modes of OH groups (i.e., free OH groups, intramolecular, and intermolecular H-bridges of the OH groups) [34,35]. The band at ~3075 cm^−1^ is a mutual band of C-H vibrations in sp_2_ hybridization and stretching vibration of the N-H bonds in secondary amides (amide B). The stretching vibrations of C=O coupled with N–H bending vibrations seen in the amide I and amide II bands arise from N–H bending vibrations coupled with C–N stretching vibrations. Another demonstration of the triple helical collagen structure can be seen in amide III (at ~1205, 1235, and 1280 cm^−1^) together with a band at 1338 cm^−1^ [36,37].

The spectral region from 3650–3150 cm^−1^ reflects stretching vibrations of amino acids, NH_2_ bonds (amide A), and OH bonds in free and interstitial water. Crosslinking causes free −NH_2_ groups to change into −NH- groups, water bonded to collagen is lost [4], and as a consequence, the integral absorbance of amide A decreases.

The formation of new isopeptide covalent bonds increases amide I absorbance. The area ratio of amide A/amide I (Figure 12B) can be used as a collagen crosslinking scale [38]. A lower A/I ratio indicates that a higher portion of collagen has crosslinked. The integrity of the collagen triple helical structure on the tertiary level can be evaluated by the ratios of the amide III band intensity (1235 cm^−1^) to the 1450 cm^−1^ band [39] (see Figure 12C).

**Figure 14:**
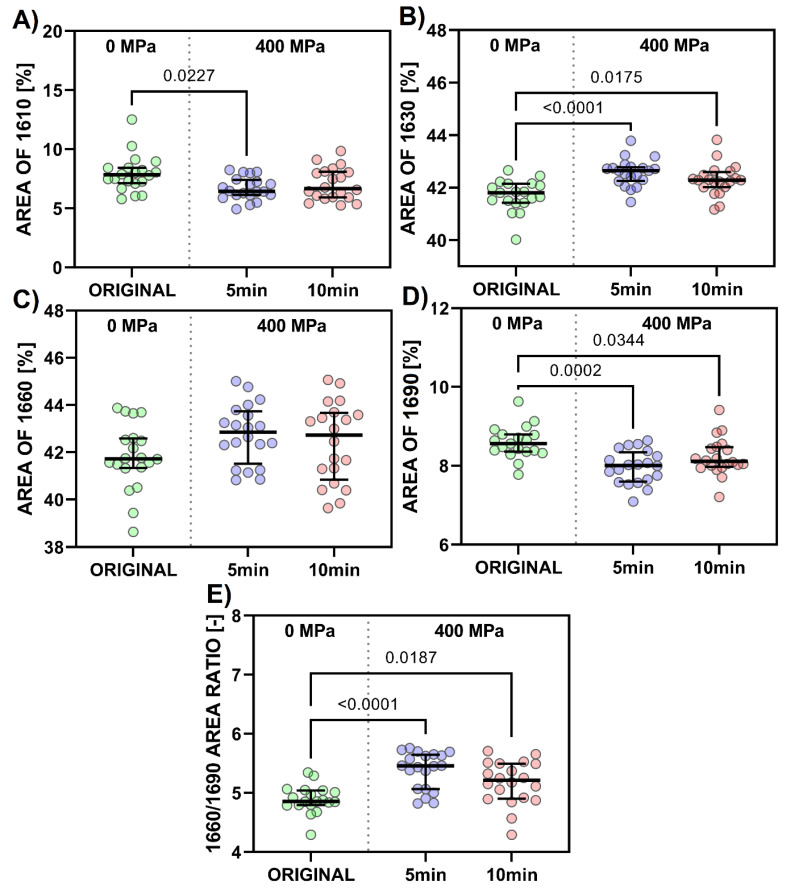
Scatter plot of (**A**) “AREA 1610,” (**B**) “AREA 1630,” (**C**) “AREA 1660,” (**D**) “AREA 1690,” and (**E**) “AREA RATIO 1660/1690” with medians and interquartile range. Note that *p*-values less than or equal to 0.05 (Dunn’s multiple comparison test; *n* = 20) are displayed for comparisons of the mean rank of each data set with the mean rank of every other data set.

Evaluation of changes in the collagen secondary structure, which can occur after applying pressure, can be better seen using a deconvolution procedure for the amide I band [40]. Four deconvoluted bands (i.e., at ~1610, 1630, 1660, and 1690 cm^−1^) were expressed as percentages of the total area amide I band and statistically analyzed (Figure 14).

### 3.7. Amino Acids Analysis

**Figure 15:**
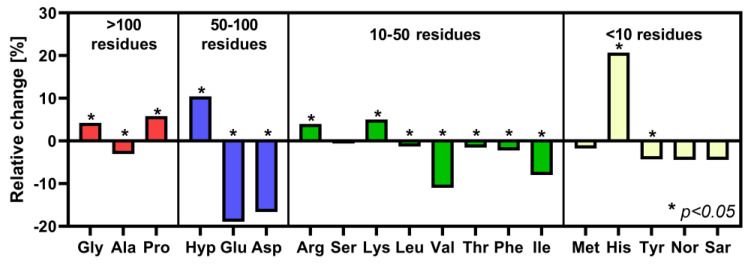
Pressure-related changes in amino acid content (number of amino acid residues per 1000 amino acid units) of collagen after 400 MPa for 10 min. Relative changes were calculated as the difference of the mean value (arithmetical mean, *n* = 6) of each amino acid content before and after pressure application. Mann–Whitney test was used to compare amino acid composition before and after pressure application (* denotes *p*-values ≤ 0.05).

Changes in amino acid composition related to UHP at 400 MPa for 10 min are shown in Figure 15. Changes in the amino acid composition after pressure application were insignificant compared to different processing types. The most striking changes were visible with glutamic (Glu) acid, aspartic (Asp) acid, and histidine (His), with changes of up to 20%. Glu and Asp were present at 50–100 residues per 1000 units, while His exists at very low concentrations (i.e., 10 residues per 1000 units). While Asp, Glu, valine (Val), and isoleucine (Ile) show a decrease, glycine (Gly), proline (Pro), hydroxyproline (Hyp), and His show an increase.

### 3.8. Scanning Electron Microscopy and the Characterization of Collagen Fibril Orientation

**Figure 16:**
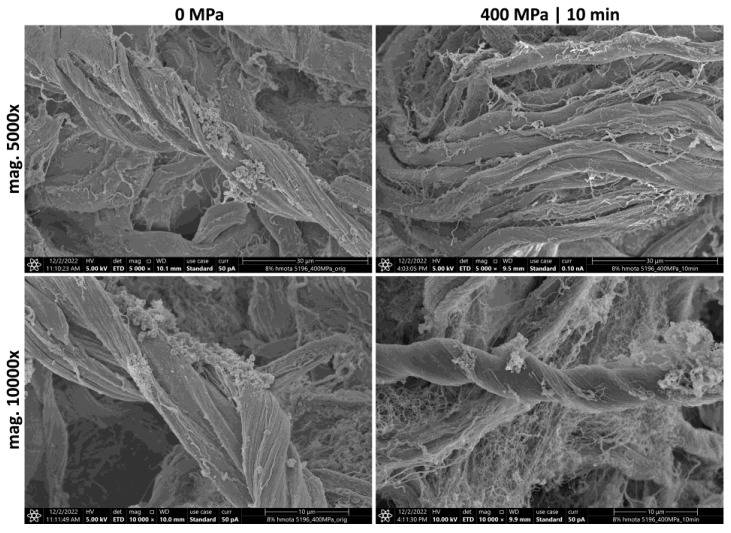
Representative SEM images of the two collagenous samples (0 MPa and 400 MPa for 10 min); upper line mag. 5000×, bar 30 μm; bottom line mag. 10,000×, bar 10 μm.

The SEM images were used to evaluate collagen fibrils’ orientation and for the qualitative evaluation of changes in ordering over longer distances after pressure application (Figure 16). No changes were seen; pressure-treated samples were typical for collagenous isolates without significant changes in structure.

## 4. Discussion

No rheological properties of UHP-treated bovine collagen measured in the small-amplitude linear viscoelastic region were found in the existing literature. Additionally, no mechanical properties of UHP-treated bovine collagen, measured by compressing collagen cylinders between two plates, or thermal properties of high-pressure-treated bovine collagen were found in the literature. However, these properties were found for porcine collagen (Potekhin et al. [22]) and bullfrog skin collagen (Nan et al. [23]).

Potekhin’s team measured thermal properties at pressures up to 200 MPa acting directly in a DSC calorimeter. They evaluated the mean temperature at denaturation transition T_m_ and denaturation energy ΔH. The T_m_ parameter increases with increasing pressure following a non-linear equation (p is pressure in MPa, T_m_ in °C), as follows:

T_m_ = 41.4 + 4.7 ⋅10^−2^ · p − 6.6⋅10^−5^ ⋅ p^2^

This equation predicts a change in the mean temperature during collagen denaturation from 41.4 °C to 47.7 °C. The parameter T_m_ can be compared with the data T_peak_ in Figure 9. Both the T_peak_ valid for the denaturation of frog collagen and the T_peak_ valid for bovine collagen increase with increasing pressure.

The denaturation energy ΔH, as presented in [22], decreases slightly with increasing pressure by about 5%. Our data for this parameter are presented in Figure 11. No downward trend can be established for pressures up to 200 MPa.

Nan et al. [23] treated bullfrog skin collagen; the study aimed to determine the effect of ultra-high pressure on the structure and properties of collagen. Native collagen extracted from bullfrog skin was processed under different ultra-high pressure treatment conditions of 300, 400, and 500 MPa. The samples were prepared as solutions in acetic acid. The pressure medium was water, and the samples were treated at 10 °C for 15 min. After pressurization, the samples were frozen and lyophilized. Before thermal analysis using the DSC method, the samples were reconstituted again in an acetic acid solution. For these reasons, it is impossible to compare the absolute values of our measurements with the results of their work [23]; instead, we can compare only the trends of dependence on the applied pressures.

The mean denaturation temperature varied from 35.4 °C for the non-pressurized collagen solution to 36.2 °C for the collagen solution pressurized to 500 MPa. The effect of pressure from 300 to 500 MPa was statistically inconclusive. The mean denaturation temperatures determined in our tests range from 35.3 to almost 36 °C, as seen in Figure 10. These data show excellent agreement, despite the different origins of the collagen samples.

The denaturation enthalpy ΔH depends on the applied pressure value from 1.4 J/g for the unpressurized sample, then 1.9 J/g for 300 MPa, 1.1 J/g for 400 MPa, and 0.9 J/g for 500 MPa. The heat-treated sample had a value of ΔH = 0.5 J/g. It is clear from these data that the value of the denaturation enthalpy has a maximum of 300 MPa, but as pressure increases, there is a significant decrease in the denaturation energy.

If we compare our data in Figure 11, we find a statistically significant increase from the untreated sample to the sample treated at a pressure of 200 MPa, but only for a holding time of 5 min. An increase in pressure to 300 MPa with a holding time of 10 min caused the ΔH value to reach a maximum. If a higher pressure was applied (i.e., 400 MPa), there was a decrease in the average value, although it is statistically inconclusive. Our values are considerably higher than those of the cited authors. The average value of ΔH reached approximately 4 J/g, whereas the average value for frog collagen was 1.3 J/g. The different sample origins could cause this almost threefold difference, but it is most likely caused by the different methods used to prepare the dry matter (i.e., the use of acetic acid solutions). It appears that the trends of dependence on the applied pressure are similar.

Gauza-Włodarczyk et al. [41] studied the thermal properties of fish and beef collagen using the DSC method. Bovine collagen was prepared from the Achilles tendon and was used in a dry state. The denaturation temperature was 220 °C. This value is quite different from the values we found for bovine collagen. The reason may be that our collagen was in its natural state and had a dry weight of 7.44% compared to the dry state used by the authors of the cited work.

Zhang et al. [42] studied bovine collagen and its properties, including denaturation temperatures, which reached a value of 37.5 °C. This temperature was very close to our measurements.

Lin and Cheng Liu [43] studied type I collagen from bird feet (BF), bovine skin (BS), frog skin (FS), porcine skin (PS), and shark skin (SS) and compared their thermal stability. The thermal transition temperatures of type I collagen from different animals decreased as shown: BF > BS > PS > FS > SS. PS collagen had a higher extractable uronic acid/protein ratio and the lowest enzymatic sensitivity. In summary, collagen BF had a higher value of hydroxyproline (Hyp) + proline (Pro) and showed higher thermal stability; PS collagen contained a more significant amount of glycosaminoglycan, resulting in high enzyme resistance. However, BF and PS collagen should be used in biomaterials due to their better biostability.

As seen in Figure 13, collagen spectra after 400 MPa in a gel state do not show significant changes compared to the original states, regardless of the holding time. A/I ratios show no statistically significant differences, with the greater data scattering representing local inhomogeneities (Figure 12B). The degree of crosslinking is comparable in all cases. Intensity ratios of amide III/1450 cm^−1^ ranged from 0.97 to 1.15; these values correspond to collagen, while a ratio around 0.76 is typical for gelatin [44]. No statistically significant differences were found (Figure 12C). Collagen’s integrity, in gels, was not damaged even after 400 MPa for 5 and 10 min. Collagen’s triple helix structure, represented by the main band at 1660, can be used as a marker of collagen change. No statistically significant differences were determined in this area (Figure 14C). Changes in other parameters range from 1 to 2%. The 1610 spectral band (Figure 14A) can be assigned to the spectral manifestation of aromatic amino acids, which may be more spectroscopically active in disintegrated states of collagen (i.e., gelatin) [45]. Band 1630 represents a denatured state of a collagen left-handed 3–10 helix (Figure 14B), and band 1690 (Figure 14D) represents β-turn and antiparallel β-sheet structures [40]. Beta sheets consist of β-strands (chains are typically 3–10 amino acids long) that are connected laterally by hydrogen bonds, thus forming a twisted, pleated sheet. Two sub-bands in the amide I spectral peak, 1660 and 1690 cm^−1^, are of particular interest (Figure 14E).

The 1660/1690 ratio can be used to evaluate mature and immature collagen crosslinks [46]. These crosslinks are present naturally in collagen and can be strongly influenced by parameters of the source animal (genus, sex, age). Figure 14E shows that this ratio does not decrease after applying high pressure. Thus, there is no damage to the mature crosslinks initially present in the analyzed collagen matrix since mature trivalent interfibrillar crosslinks present in collagen are generally more resistant to various types of attack (chemical, thermal, radiation, etc.). Production of mature crosslinks starts at birth and gradually increases with age [47]. It has been shown before [41,48] that mammalian collagen (e.g., bovine) is more stable than marine collagen. Our conclusions correlate with the published results of Nan et al. [23], who studied the effect of high pressure on bullfrog collagen. They concluded that at low pressures (up to 400 MPa), the perpendicular pressure to the collagen axis dominates and leads to a tightening of the triple helix, while at high pressure (>400 MPa), forces acting parallel to the collagen axis dominate and triple helix tends to dissociate like a zipper. Heremans and Smeller [49] state that high pressure rarely affects covalent bonds, and even α-helix and β-sheet structures appear almost incompressible. In aqueous solutions, pressure mainly affects tertiary and quaternary protein structures.

Our amino acids analysis cannot be compared with literature data because, to our knowledge, amino acid composition changes after high-pressure bovine collagen treatment have not been published. It is only possible to find scientific works that deal with the change in the concentration of amino acids in meat or other protein products after applying pressure, but not in collagen materials. These products contain a whole range of proteins, including non-collagenous ones, so these results are difficult to compare. The amino acid triplet Gly, Pro, and Hyp form repetitive sequences in collagen, and His shows an increase, therefore showing resistance to pressure. Hyp increases the stability of the collagenous triple-helix. The unique triple-helical motif (Gly-Pro-Hyp) is responsible for collagen higher-order assembly and mechanical strength [50]. Histidine can be a part of mature crosslinks, which show high stability and resistance to various treatments [47]. An increase in His concentration was also shown by Ahmed et al. [51], following high-pressure treatment at 375 MPa for 20 min. The increase is apparently caused as compensation for the decrease in other amino acids.

In the absence of chemical agents or the formation of radicals by radiolysis, the conversion of amino acids into each other is unlikely. The Glu, Asp, Val, and Ile belong to aliphatic amino acids, show decreased concentration, and are more sensitive to UHP. The side chains of Glu and Asp have carboxylic acid groups whose pKas are low enough to lose protons, becoming negatively charged. Appropriate amounts of UHP cycling can disrupt hydrogen bonds, ionic bonds, and disulfide bonds [52]. Opposite this, Val and Ile are non-polar with long carbon chains; consequently, they are hydrophobic. UHP strongly modifies the structure of water, which allows exposure of the non-polar surface to water and eventually completely stops hydrophobic interactions resulting in the reorganization of amino acid residues in peptide chains [52]. SEM microscopy showed that 400 MPa for 10 min did not evoke significant changes in configuration and ordering over longer distances than the original collagenous gel. As shown by our previous studies [29,46], fibril bundle orientation (i.e., the tertiary and quaternary structure of collagen) plays a significant role in the mechanical properties of gels.

## 5. Conclusions

### 5.1. Rheological Properties

The viscoelastic properties of the measured bovine collagen sample can be characterized as the area of linear viscoelasticity at small oscillation amplitudes using the parameters G′ and G′′ for the measurement temperature of 10 °C and pH 2.13. The parameters G′ and G′′ increase with increasing frequency and the angular velocity of oscillations, respectively.

The elastic modulus G′ can be characterized by a simplified combined Kelvin–Voigt–Maxwell model (see Equation (3)). This equation statistically convincingly describes the experimental data valid for a given collagen sample. We tried to correlate the parameters of the simplified Kelvin–Voigt–Maxwell model G_1_ and G_2_ using Equations (5) and (6). It has been shown that these parameters do not depend statistically conclusively on the pressure or hold times in the range of the values used for these process parameters. The G_1_ value can be characterized by the constant a_1_ = 9804.4 Pa. The G_2_ value can be characterized by the constant d_2_ = 2246.5 Pa.

### 5.2. Mechanical Properties

The dependence of the stress Ϭ on the relative deformation ϵ can be described very well using Equation (2). The regression line representing this equation fits well with the experimental data. The effect of pressure on the elastic modulus measured by compressing cylinder-shaped collagen samples was statistically negligible.

### 5.3. Thermal Properties

Using a high-quality apparatus for determining the thermal properties of collagen, working on the principle of differential scanning calorimetry, numerical values of these properties were determined for collagen samples under different pressures.

By analyzing the variance of all experimental values, it was determined that the temperature at the beginning of the thermal reaction caused by the heating of the T_on_ collagen sample was statistically demonstrably dependent on the pressure and holding time. The ΔH parameter showed the same dependence. This parameter represents the area under the peak of the curve, which characterizes the energy required for the ongoing reaction caused by heating the collagen sample.

The T_peak_ parameter shows a statistically significant dependence on the pressure but is independent of holding time. The H_peak_ parameter, on the other hand, does not depend on pressure but statistically significantly depends on holding time.

### 5.4. Overall Rating, Limitations, and Practical Implications

The rheological properties measured as the area of linear viscoelasticity were not changed statistically significantly due to the influence of pressure or holding time. In addition, the mechanical properties measured by compression between two plates were not statistically significantly influenced by either the pressure or the hold time. The thermal properties T_on_ and ΔH measured using differential calorimetry depend on both pressure and hold time.

The results of amino acids and FTIR analysis showed that the exposure of the collagenous gels to high pressure of 400 MPa, regardless of hold time (5 and 10 min), caused only minor changes in the primary and secondary structure while preserving collagenous polymeric integrity. SEM analysis did not show changes in ordering and collagen fibrils orientation over longer distances after 400 MPa for 10 min.

As known from the literature (e.g., [20]), UHP inactivates living microorganisms, a wide range of food pathogens, and endogenous enzymes that cause spoilage and extends shelf-life with a minimum impact on sensory and other quality parameters. This technology can be used for microbial stabilization of collagen without significant changes in its properties, as shown in this paper.

However, the results from this study relate to bovine collagen treated with 400 MPa for 10 min and cannot be applied to collagen in general. Collagen can be extracted from various animal sources and such natural material embodies high inhomogeneity. Collagen integrity, structure stability, and resistance to various treatments (chemical, thermal, irradiation, and UHP) are influenced by animal genus, sex, age, breeding, and so on, as well as extraction methods and other treatments.

There is a limit related to the source of collagen—only bovine collagen has a high mass fraction. The rheological properties are limited only to the linear viscoelasticity and range of used deformations. Mechanical properties were measured up to the limited allowable deformation.

Ultra-high-pressure (UHP) treatment is a technology applicable to food and biomaterial preservation. As shown by many publications, industry applications already exist. With any treatment, there is always a potential risk of adverse effects on the properties of treated materials. Our research confirmed that using UHP does not change the essential properties of treated products.

We found no changes in rheological properties affecting the transport or subsequent treatment of the product. No special or additional changes to the mechanical apparatuses are required. Since the mechanical properties of the product are not changed by UHP treatment, there are no reasons to expect changes in sensory properties concerning consumability (chewing and swallowing). Treated products can withstand the same mechanical load before and after UHP treatment. Thermal properties exhibited a change after UHP treatment, which must be considered before thermal treatment of the product.

## Figures and Tables

**Table 1:** Numerical values of the parameters of Equation (4) depending on the pressure treatment parameters.

Pressure Holding Time (min)	Pressure (MPa)	G_1_ (Pa)	G_2_ (Pa)	τ_1_ (s)	τ_2_ (s)	µ_1_ (Pa∙s)	µ_2_ (Pa∙s)	r^2^ (−)	r (−)
0	0	9105	2089	5.40	0.20	49,206	11,292	0.588	0.767
5	200	12,111	2856	5.03	0.19	60,973	14,379	0.700	0.837
10	200	10,931	2516	5.20	0.20	568,41	13,083	0.229	0.479
5	300	10,006	2300	5.04	0.20	50,393	11,586	0.628	0.793
10	300	10,464	2453	4.98	0.20	52,116	12,217	0.519	0.720
5	400	11,465	2625	5.16	0.20	59,112	13,534	0.793	0.891
10	400	10,519	2518	5.94	0.21	62,468	14,951	0.610	0.781

**Table 2:** Parameter values of Equation (5).

Parameter	Value	Standard Deviation	t-Parameter	*p*	Evaluation
a_1_ (Pa)	9804.4	955.1	10.3	0.0005	significant
b_1_ (Pa/min)	28.7	145.7	0.2	0.854	insignificant
c_1_ (−)	2.6	3.9	0.7	0.545	insignificant

**Table 3:** Parameter values of Equation (6).

Parameter	Value	Standard Deviation	t-Parameter	*p*	Evaluation
d_2_ (Pa)	2246.5	230.9	9.7	0.0006	significant
e_2_ (Pa/min)	11.9	35.2	0.3	0.752	insignificant
f_2_ (−)	0.6	0.95	0.6	0.557	insignificant

**Table 4:** Parameters of the simplified HGO model. Values with 95% confidence bounds.

Pressure (MPa)	Holding Time (min)	*E* (kPa)	*k*_1_ (kPa)	r^2^ (−)
0	0	15.63 ± 0.69	−2.01 ± 0.63	0.945
200	5	17.95 ± 0.73	−2.51 ± 0.64	0.949
	10	16.84 ± 0.45	−1.89 ± 0.40	0.980
300	5	17.54 ± 0.83	−2.95 ± 0.72	0.919
	10	17.04 ± 0.69	−3.03 ± 0.60	0.941
400	5	18.01 ± 0.58	−3.06 ± 0.50	0.964
	10	18.17 ± 0.52	−3.52 ± 0.45	0.968

**Table 5:** Results of an analysis of variance of thermal properties of high-pressure-treated collagen.

Thermal Properties/ Parameters of UHP	T_on_ (°C)		T_peak_ (°C)		H_peak_ (J/g∙ K)		ΔH (J/g)	
	*p*	Statistical Significance	*p*	Statistical Significance	*p*	Statistical Significance	*p*	Statistical Significance
Pressure (MPa)	0.00210	yes	0.00358	yes	0.05608	no	0.00045	yes
Holding time (min)	0.02155	yes	0.14404	no	0.04463	yes	0.00399	yes

Note: if parameter *p* is less than 0.05, the property is statistically significantly dependent on the tested process parameter with a probability of 95%.

## Data Availability

All experimental data are available from the authors upon written request.

## References

[1] Lasek W. (1978). Kolagen.

[2] Owczarzy A., Kurasiński R., Kulig K., Rogóż W., Szkudlarek A., Maciążek-Jurczyk M. (2020). Collagen—Structure, properties and Applications. Eng. Biomater..

[3] Shoulders M.D., Raines R.T. (2009). Collagen Structure and Stability. Annu. Rev. Biochem..

[4] Sionkowska A., Skrzyński S., Śmiechowski K., Kołodziejczak A. (2017). The review of versatile application of collagen. Polym. Adv. Technol..

[5] Liu J., Hua F., Zhang H., Hu J. (2023). Influence of using collagen on the soft and hard tissue outcomes of immediate dental implant placement: A systematic review and meta-analysis. J. Stomatol. Oral Maxillofac. Surg..

[6] Hyder P.R., Dowell P., Singh G., Dolby A.E. (1992). Freeze-Dried, Crosslinked Bovine Type I Collagen: Analysis of Properties. J. Periodontol..

[7] Brum I.S., Elias C.N., de Carvalho J.J., Pires J.L.S., Pereira M.J.S., de Biasi R.S. (2021). Properties of a bovine collagen type I membrane for guided bone regeneration Applications. e-Polymers.

[8] Yu L., Cavelier S., Hannon B., Wei M. (2023). Recent development in multizonal scaffolds for osteochondral regeneration. Bioact. Mater..

[9] Liu W., Ma Z., Wang Y., Yang J. (2023). Multiple nano-drug delivery systems for intervertebral disc degeneration: Current status and future perspectives. Bioact. Mater..

[10] Tang C., Zhou K., Zhu Y., Zhang W., Xie Y., Wang Z., Zhou H., Yang T., Zhang Q., Xu B. (2022). Collagen and its derivatives: From structure and properties to their applications in food industry. Food Hydrocoll..

[11] Messens W., Van Camp J., Huyghebaert A. (1997). The use of high pressure to modify the functionality of food proteins. Trends Food Sci. Technol..

[12] Knorr D., Heinz V., Buckow R. (2006). High pressure application for food biopolymers. Biochim. Biophys. Acta.

[13] Dumay E., Picart L., Regnault S., Thiebaud M. (2006). High pressure–low temperature processing of food proteins. Biochim. Biophys. Acta.

[14] Tao Y., Sun D.W., Hogan E., Kelly A.L., Sun D.W. (2014). Chapter 1—High-Pressure Processing of Foods: An Overview. Emerging Technologies for Food Processing.

[15] Chen L., Ma L., Zhou M., Liu Y., Zhang Y. (2014). Effects of pressure on gelatinization of collagen and properties of extracted gelatins. Food Hydrocoll..

[16] Mobasheri A., Mahmoudian A., Kalvaityte U., Uzieliene I., Larder C.E., Iskandar M.M., Kubow S., Hamdan P.C., de Almeida C.S., Favazzo L.J. (2021). White Paper on Collagen Hydrolyzates and Ultrahydrolyzates: Potential Supplements to Support Joint Health in Osteoarthritis?. Curr. Rheumatol. Rep..

[17] Ma Y., Teng A., Zhao K., Zhang K., Zhao H., Duan S., Li S., Guo Y., Wang W. (2020). A top-down approach to improve collagen film’s performance: The comparisons of macro, micro and nano sized fibers. Food Chem..

[18] Cheftel J.C., Balny C., Hayashi R., Heremans K., Masson P. (1992). Effects of high hydrostatic pressure on food constituents: An overview. High Pressure and Biotechnology.

[19] Balny C., Masson P. (1993). Effects of high pressure on proteins. Food Rev. Int..

[20] Tauscher B. (1995). Pasteurization of food by hydrostatic high pressure: Chemical aspects. Z. Für Lebensm. -Unters. Und Forsch..

[21] Gómez-Guillén M.C., Giménez B., López-Caballero M.E., Montero M.P. (2011). Functional and bioactive properties of collagen and gelatin from alternative sources: A review. Food Hydrocoll..

[22] Potekhin S.A., Senin A.A., Abdurakhmanov N.N., Tiktopulo E.I. (2009). High pressure stabilization of collagen structure. Biochim. Et Biophys. Acta.

[23] Nan J., Zou M., Wang H., Xu C., Zhang J., Wei B., He L., Xu Y. (2018). Effect of ultra-high pressure on molecular structure and properties of bullfrog skin collagen. Int. J. Biol. Macromol..

[24] Landfeld A., Houška M., Skočilas J., Žitný R., Novotná P., Štancl J., Dostál M., Chvátil D. (2016). The Effect of Irradiation on Rheological and Electrical Properties of Collagen. Appl. Rheol..

[25] Holzapfel G.A., Gasser T.C., Ogden R. (2004). Comparison of a Multi-Layer Structural Model for Arterial Walls with a Fung-Type Model, and Issues of Material Stability. J. Biomech. Eng..

[26] Chlup H., Skočilas J., Štancl J., Houška M., Žitný R. (2022). Effects of extrusion and irradiation on the mechanical properties of a water-collagen solution. Polymers.

[27] Bella J., Brodsky B., Berman H.M. (1995). Hydration structure of a collagen peptide. Structure.

[28] https://www.agilent.com/cs/library/applications/compendium-%20aminoacid-advancebio-5994-0033EN-us-agilent.pdf.

[29] Palay S.L., McGee-Russell S.M., Gordon S., Grillo M.A. (1962). Fixation of neural tissue for electron microscopy by perfusion with solution of osmium tetroxide. J. Cell Biol..

[30] Barnes H.A. (2000). A Handbook of Elementary Rheology.

[31] Šupová M., Suchý T., Chlup H., Štípek J., Žitný R., Landfeld A., Skočilas J., Žaloudková M., Rýglová Š., Braun M. (2023). The comprehensive evaluation of two collagen gels used for sausage casing extrusion purposes: The role of the structural and mechanical properties. J. Food Eng..

[32] Štěpánek V. Matematická statistika v chemii, Státní nakladatelství technické literatury, Praha 1975.

[33] Riaz T., Zeeshan R., Zarif F., Ilyas K., Muhammad N., Safi S.Z., Rahim A., Rizvi S.A.A., Rehman I.U. (2018). FTIR analysis of natural and synthetic collagen. Appl. Spectrosc. Rev..

[34] Szymanski H.A., Erickson R.E. (1970). Infrared Band Handbook.

[35] Coates J., Meyers R.A. (2000). Interpretation of Infrared Spectra, a Practical Approach. Encyclopedia of Analytical Chemistry.

[36] Jackson M., Choo L.P., Watson P.H., Halliday W.C., Mantsch H.H. (1995). Beware of connective tissue proteins: Assignment and implications of collagen absorptions in infrared spectra of human tissues. Biochim. Biophys. Acta.

[37] Rabotyagova O.S., Cebe P., Kaplan D.L. (2008). Collagen Structural Hierarchy and Susceptibility to Degradation by Ultraviolet Radiation. Mater. Sci. Eng. C.

[38] Sommer A., Dederko-Kantowicz P., Staroszczyk H., Sommer S., Michalec M. (2021). Enzymatic and Chemical Crosslinking of Bacterial Cellulose/Fish Collagen Composites—A Comparative Study. Int. J. Mol. Sci..

[39] Figueiro S., Goes J., Moreira R., Sombra A. (2004). On the physicochemical and dielectric properties of glutaraldehyde crosslinked galactomannan–collagen films. Carbohydr. Polym..

[40] Payne K.J., Veis A. (1988). Fourier transform IR spectroscopy of collagen and gelatin solutions: Deconvolution of the amide I band for conformational studies. Biopolymers.

[41] Gauza-Włodarczyk M., Kubisz L., Mielcarek S., Włodarczyk D. (2017). Comparison of thermal properties of fish collagen and bovine collagen in the temperature range 298–670 K. Mater. Sci. Eng. C.

[42] Zhang Z., Li G., Shi B. (2006). Physicochemical properties of collagen, gelatin and collagen hydrolysate derived from bovine limed split wastes. J. Soc. Leather Technol. Chem..

[43] Lin Y.K., Liu D.C. (2006). Comparison of physical–chemical properties of type I collagen from different species. Food Chem..

[44] Zhang X., Xu L., Huang X., Wei S., Zhai M. (2012). Structural study and preliminary biological evaluation on the collagen hydrogel crosslinked by γ-irradiation. J. Biomed. Mater. Res. A.

[45] Prystupa D.A., Donald A.M. (1996). Infrared study of gelatin conformations in the gel and sol states. Polym. Gels Netw..

[46] Sanden K.W., Böcker U., Ofstad R., Pedersen M.E., Høst V., Afseth N.K., Rønning S.B., Pleshko N. (2021). Characterization of collagen structure in normal, wooden breast and spaghetti meat chicken fillets by FTIR microspectroscopy and histology. Foods.

[47] Gaar J., Naffa R., Brimble M. (2020). Enzymatic and non-enzymatic crosslinks found in collagen and elastin and their chemical synthesis. Org. Chem. Front..

[48] Gauza-Włodarczyk M., Kubisz K., Włodarczyk D. (2017). Amino acid composition in determination of collagen origin and assessment of physical factors effects. Int. J. Biol. Macromol..

[49] Heremans R., Smeller L. (1998). Protein structure and dynamics at high pressure. Biochim. Biophys. Acta.

[50] Zitnay J.L., Li Y., Qin Z., San B.H., Depalle B., Reese S.P., Buehler M.J., Yu S.M., Weiss J.A. (2017). Molecular level detection and localization of mechanical damage in collagen enabled by collagen hybridizing peptides. Nat. Commun..

[51] Ahmed J., Habeebullah S.F.K., Alagarsamy S., Mulla M.Z., Thomas L. (2022). Impact of High-Pressure Treatment on Amino Acid Profile, Fatty Acid Compositions, and Texture of Yellowfin Seabream (*Acanthopagrus arabicus*) Filets. Front. Sustain. Food Syst..

[52] Li X., He Z., Xu J., Su C., Xiao X., Zhang L., Zhang H., Li H. (2022). Conformational Changes in Proteins Caused by High-Pressure Homogenization Promote Nanoparticle Formation in Natural Bone Aqueous Suspension. Foods.

